# Seroprevalence of Leptospiral Antibodies in Canine Population in and around Namakkal

**DOI:** 10.1155/2013/971810

**Published:** 2013-07-09

**Authors:** N. R. Senthil, K. M. Palanivel, R. Rishikesavan

**Affiliations:** Department of Veterinary Epidemiology and Preventive Medicine, Veterinary College and Research Institute and Tamilnadu Veterinary and Animal Sciences University, Tamilnadu, Namakkal 637 002, India

## Abstract

Leptospirosis is a reemerging and a complex zoonotic bacterial disease, caused by pathogenic serovars of *Leptospira interrogans.* A total of 124 sera samples of dogs belonging to different categories like vaccinated, unvaccinated-semiowned, and stray dogs were subjected to sampling. Microscopic agglutination test (MAT) was conducted by using *Leptospira* culture. Out of 42 vaccinated dogs, 24 (57%) were positive to one or more serovars. Of the 24, 22 (52.3%), 11 (26.19%), 4 (9.5%), 1 (3%), and 2 (4.7%) were positive to *icterohaemorrhagiae, canicola, pomona, grippotyphosa,* and *autumnalis*, respectively. Of the 48 unvaccinated semiowned dogs, 10 (28.8%) showed positive agglutination to one or more serovars. Of the 10 samples, 7 (14.5%), 2 (4.1%), 3 (6.2%), 3 (6.2%), and 5 (10.2%) were positive to *icterohaemorrhagiae, canicola, pomona, grippotyphosa,* and *autumnalis,* respectively. Among the 34 stray dogs, 12 showed positive agglutination to one or more leptospiral antibodies. Of the 12 samples, 6 (17.6%) showed positive agglutination to *icterohaemorrhagiae,* 2 (5.8%) to *canicola*, 5 (14.7%) to *pomona*, 7 (20.5%) to *grippotyphosa,* and 5 (4.7%) to *autumnalis*. This study emphasized the changing trends in the epidemiology of leptospirosis with higher prevalence of serovar *L. grippotyphosa* in street dogs.

## 1. Introduction

The five leptospiral serovars known to be endemic in and around Namakkal, Tamilnadu, are *L. interrogans* serovars *icterohaemorrhagiae, canicola, pomona, grippotyphosa,* and *autumnalis*. Exposure to leptospira organisms is common in dogs reported by [1–3]. Currently available leptospiral vaccines for dogs in India contain inactivated *Leptospira interrogans* serovars *icterohaemorrhagiae *and *canicola* [4] which are antigenically similar to serovar *copenhageni* being from the same serovars *icterohaemorrhagiae *[5] and will stimulate active immunity to both serovars. A serosurveillance study was conducted to provide further information on the changing epidemiological trend of canine leptospirosis infections in Tamilnadu. The aim in the present study was to investigate the prevalence of serum antibodies against five endemic leptospiral serovars in dogs identifying the patterns of risk and generating further hypotheses for investigation of canine leptospirosis infections in Tamilnadu, India.

## 2. Materials and Methods

### 2.1. Data Collection and Handling

The study population was a convenience sample of  124 canine  serum samples submitted to the diagnostic laboratory of the Department of Veterinary Epidemiology and Preventive Medicine (DVEPMD), Veterinary College and Research Institute, Namakkal, Tamilnadu. Blood was collected in a plain vacutainer tubes and submitted to Leptospirosis laboratory (DVEPMD) for diagnostic purposes unrelated to this study. The samples were received from 8 different regions: 284 samples were from half of the Namakkal district and 174 from the same regions (Table 1) from resident dogs (vaccinated pet), resident semiowned dogs, and stray dogs (unvaccinated) in and around Namakkal. Information provided with the data included breed, sex, age, and the region the animal resided when the blood was collected and simultaneously the blood was collected from stray dogs with the help of animal attendants from the same regions randomly.

### 2.2. Microscopic Agglutination Test (MAT)

Sera were tested against five serovars most likely to cause disease in dogs in Namakkal regions which are *L. interrogans*: serovars *icterohaemorrhagiae, pomona, canicola, autumnalis,* and *grippotyphosa*. The MAT was performed as per the method of [6]. A homologous, high titred antiserum was included in each testing session. Serum dilutions were prepared in 8-well “U” bottomed disposable microtitre plates (Tarson). A serial twofold dilution of each serum was made in phosphate buffered saline (pH 7.2) starting with an initial dilution of 1 : 10. An equal volume (i.e., 50 *μ*L) of culture was added to each well, mixed by gentle rocking, and incubated at 37°C for 2 hrs after sealing with polyethylene sheet. The MAT titre was the reciprocal of the highest dilution of the serum in which >50% of the antigen was agglutinated. A minimum titre of 1 : 40 and above was taken as the positive agglutination reaction in endemic areas.

### 2.3. Data Analysis

The variable age was divided into 5 categories (1-2 yrs; 2-3; 3-4; 4-5, and 5 and above). The breeds were classified into 3 broad categories. Small breeds (Pomeranian, Poodles, Pug, Dachshund, and Spitz), larger breeds (Labrador, Great Dane, Golden retriever, German shepherd, and English mastiff), and Terrier breeds (retriever, nondescript (Mongrel), Rajapalayam, Combi, etc.) were taken for investigation and the association between prevalence of positive leptospiral titres for any serovars and protective titre of each individual were analysed.

## 3. Results and Discussion

The study population that included 460 dogs confirmed that leptospira interogans serovar *icterohaemorrhagiae* was the most common leptospiral serovars and that this population of dog had positive titre of 1 : 40. In addition, the prevalence of titres to leptospira interogans serovar icterohaemorrhagiae in dogs sampled 7.7 percent was similar to the prevalence of 9.5 percent reported by [2]. However 18.8 percent of positive cases of *L. icterohaemorrhagiae* were maintained in vaccinated dog population in this region. This could be the reason for higher prevalence of *L. icterohaemorrhagiae* in vaccinated dog population in these areas. However, if the dogs were exposed to natural infection before vaccination, naturally the antibody titres were increased and respond to given vaccine in this study. Prevalence of positive titres to *L. icterohaemorrhagiae* of 12 percent in region wise (Tamilnadu) was reported by [1]. There was a little change in the prevalence of *pomona* (1.4%), *canicola* (3.9%), *grippotyphosa* (0.4%), and *autumnalis *(0.7%) in vaccinated dogs when compared with unvaccinated dogs population: *pomona* (10.2%), *canicola* (9.1%), *grippotyphosa* (11.4%), and *autumnalis *(10.8%), respectively (Table 3). This finding is consistent with a report of [7]. The overall prevalence of any one leptospiral antibodies to *L. icterohaemorrhagiae* was 26 percent followed by 17 percent in small breeds and 14.7 percent in larger breeds. Similar findings were reported by [8, 9]. The reasons for higher prevalence of leptospira antibodies in Mongrel breeds than other breeds, thus the hypotheses that increased contact with rats and therefore having increased positive titre of leptospirosis by this survey group of the sample size in this breed group (*n* = 185) was low and may not have been sufficient to detect differences in prevalence of positive leptospiral titres by breed in the total population of 80,239 in this region.

There is an anecdotal perception among veterinarians that urban dogs are at lesser role of exposure to leptospires than other dogs. In the present study small breeds live in urban environment did not have a lesser incidence of titres to* L. interrogans* serovar *icterohaemorrhagiae* than other breeds. This finding might be due to vaccination; however vaccine induced titres rarely result in >300 and these titres only persist for 3–12 weeks after vaccination, falling below MAT titres of 1 : 100. This finding is consistent with reported data of [2, 10] that reported that dogs most likely infect natural exposure in naive or vaccinated dogs.

Vaccine induced titres against serovars *icterohaemorrhagiae* and *canicola* make interpretation of multiple positive titres and *pomona, grippotyphosa,* and *autumnalis* titres more difficult. The elevated MAT titres to leptospires reflect natural exposure and not by vaccination as reported by [11]. In this study, nonvaccinated dogs will have increased antibody response when compared to vaccinated dogs. The higher antibody prevalence of serovars *grippotyphosa, autumnalis, pomona, *and *canicola* in this study may reflect a population of vaccinated dogs responding to natural challenge, rather than increase in titres after natural infection unrelated to vaccine administration [12, 13].

There was statistically significant difference in prevalence of positive leptospiral titres between the vaccinated and non vaccinated dogs. The reason for increased leptospiral antibody titers might be the changing epidemiology of canine leptospirosis. The changes include increased incidence or recognition of clinical disease caused by serovars not currently included in commercially available canine vaccines and may also be due to contact with wild and livestock reservoir hosts.

Dogs aged 5 years or older had a significant reduced prevalence of positive titres to leptospiral serovars when compared to dogs less than 5 years of age. There was a positive association that could be made with both sexes (male or female) and the presence of a leptospiral MAT titre of ≥96 (Table 2). This finding is in contrast to other reports which showed significantly higher titre in male dogs which were thought to be more likely to roam and therefore be exposed to infection [14].

The titre value of 1 : 100 or greater was considered as positive for leptospirosis [2]. For this study we recorded titres of 1 : 40 and above considered as positive. This cut-off will increase specificity of the positive results thus making conclusions regarding factors associated with the prevalence of positive leptospiral titres more compelling. There is no variability in titres reported by different laboratories testing identical samples [15].

The prevalence of higher leptospiral antibodies in canine population indicated that testing for multiple serovars is known to be circulating in the local canine population especially in the diagnosis of acute disease. Similarly, [16, 17] also found that multiple serovars are circulating in vaccinated and non vaccinated canine population throughout the world.

Generally, vaccination against leptospirosis has been recommended for dogs, because of the prevalence of serovars *icterohaemorrhagiae* and *canicola* in rat population [1]. No nationwide or even statewide surveys on canine leptospirosis or maintenance host have been conducted since then. This study supports the conclusion that exposure to serovars *grippotyphosa* and *autumnalis *is common to household dogs rather than not present in this region and should be considered as a component of vaccines used in dogs. Where these serovars are known to be prevalent inclusion of serovars *pomona, grippotyphosa,* and *autumnalis* as part of canine leptospirosis vaccine should be considered for dogs of pure breed or nondescript mongrel at increased risk of exposure to this serovars.

The estimates on the population at risk were obtained from records on numbers of registered dogs from the veterinary dispensary, the National Animal Census 2007, Department of Animal Husbandry and Fisheries, Government of India; these estimates are based on the number of registered dogs in the veterinary dispensaries will provide the estimates of the proportion of the population at risk sampled for this study is likely to be less than stated in this survey.

The samples included in this study were collected over a one-year period during summer and winter. Secondary rainfall variations affecting survivability and transmission of leptospires, in combination with a short duration of titres after exposure, may have confounded these results. However, the summer and winter months in Tamilnadu typically have very different rainfalls, and the sampling period could be considered to cover the lowest risk period and the highest risk period of warm, wet weather. Further studies could more worthwhile for examining the seasonal variations in exposure.

## Figures and Tables

**Table 1:** Estimate of dog population at risk and number of sera samples per 10,000 dogs at risk population for each region of Namakkal district from a survey of MAT titres to leptospires, total: 18, 39,791.

Place	No. of sampled	Estimated population at risk	No. of sampled per 10,000 population
Vaccinated			
Erumaipatti	39	4200	92.8
Mohanur	31	3600	86.1
Namagiripet	35	2800	125.0
Namakkal town	56	6000	93.3
Puduchatram	27	2400	112.5
Rasipuram	45	5000	90.0
Sendamangalam	25	2000	125.0
Vennandur	26	1700	152.9
**Total**	**284**	**27,700**	**102.5**

Unvaccinated			
Erumaipatti	21	1100	190.9
Mohanur	19	1400	135.7
Namagiripet	21	1500	140.0
Namakkal town	32	2200	145.4
Puduchatram	19	1000	190.0
Rasipuram	26	1800	144.4
Sendamangalam	18	1200	150.0
Vennandur	20	800	250.0
**Total**	**176**	**11,000**	**160.0**

Population at risk data obtained from Veterinary Dispensaries and Regional Animal Disease Intelligence Unit Survey (2001).

**Table 2:** Number and percentage of each variable with MAT titre of >90 for any one of leptospira serovars.

Variable	Level	Number (% of study population)	Positive MAT (% of level)
Age	1-2 years	80 (17.4)	21 (26.3)
	2-3 years	90 (19.6)	26 (28.9)
	3-4 years	86 (18.7)	32 (37.2)
	4-5 years	94 (20.4)	18 (19.1)
	5 and above	110 (23.9)	7 (6.4)

Sex	Male	263 (57.2)	55 (20.9)
	Female	197 (42.8)	42 (21.0)

Breed size	Small breeds	112 (24.3)	19 (17.0)
	Larger breeds	143 (31.0)	21 (14.7)
	Mongrel	185 (40.2)	48 (26.0)

Vaccination status	Vaccinated	284 (61.7)	126 (44.4)
	Unvaccinated	176 (38.3)	143 (81.3)

**Table 3:** Count and prevalence of microscopic agglutination test titres >96 to individual serovars icterohaemorrhagiae, grippotyphosa, canicola, pomona, autumnalis, and any one of serovars in dogs.

Serovars	Vaccinated		Unvaccinated		Region wise	
	Count	Prevalence	Count	Prevalence	Count	Prevalence
*icterohaemorrhagiae *	22	7.7	33	18.8	55	12.0
*grippotyphosa *	1	0.4	20	11.4	21	4.6
*canicola *	11	3.9	16	9.1	27	5.9
*pomona *	4	1.4	18	10.2	22	4.8
*autumnalis *	2	0.7	19	10.8	21	4.6
Any one of serovars	53	18.7	92	52.3	145	31.5
